# Perinatal Pb^2+^ exposure alters the expression of genes related to the neurodevelopmental GABA-shift in postnatal rats

**DOI:** 10.1186/s12929-018-0450-4

**Published:** 2018-05-24

**Authors:** Lorenz S. Neuwirth, Greg R. Phillips, Abdeslem El Idrissi

**Affiliations:** 1 Department of Psychology, SUNY Old Westbury, 223 Store Hill Road, Bldg.: NAB, Room: 2059, Old Westbury, NY 11568-1700 USA; 2 SUNY Old Westbury, Neuroscience Research Institute, 223 Store Hill Road, Bldg.: NAB, Room: 2059, Old Westbury, NY 11568-1700 USA; 3 Department of Biology, The College of Staten Island (CUNY), Staten Island, NY 10314 USA; 4 The CUNY Graduate Center, Biology Program, New York, NY 10016 USA; 5 The Center for Developmental Neuroscience, Staten Island, NY 10314 USA

**Keywords:** GABA-shift, Lead exposure, Neurodevelopment, Neurotoxicant, mRNA patterns, Prefrontal cortex, Hippocampus, Postnatal development, L-type calcium channels, Frontoexecutive dysfunction

## Abstract

**Background:**

Lead (Pb^2+^) is an environmental neurotoxicant that disrupts neurodevelopment, communication, and organization through competition with Ca^2+^ signaling. How perinatal Pb^2+^ exposure affects Ca^2+^-related gene regulation remains unclear. However, Ca^2+^ activates the L-Type voltage sensitive calcium channel β-3 subunit (Ca-β3), which autoregulates neuronal excitability and plays a role in the GABA-shift from excitatory-to-inhibitory neurotransmission.

**Method:**

A total of eight females (*n* = 4 Control and *n* = 4 Perinatal) and four males (*n* = 2 Control and *n *= 2 Perinatal) rats were used as breeders to serve as Dams and Sires. The Dam’s litters each ranged from *N* = 6–10 pups per litter (*M* = 8, *SD* = 2), irrespective of Pb^2+^ treatment, with a majority of males over females. Since there were more males in each of the litters than females, to best assess and equally control for Pb^2+^− and litter-effects across all developmental time-points under study, female pups were excluded due to an insufficient sample size availability from the litter’s obtained. From the included pup litters, 24 experimentally naïve male Long Evans hooded rat pups (Control *N* = 12; Pb^2+^ *N* = 12) were used in the present study.  Brains were extracted from rat prefrontal cortex (PFC) and hippocampus (HP) at postnatal day (PND) 2, 7, 14 and 22, were homogenized in 1 mL of TRIzol reagent per 100 mg of tissue using a glass-Teflon homogenizer. Post-centrifugation, RNA was extracted with chloroform and precipitated with isopropyl alcohol. RNA samples were then re-suspended in 100 μL of DEPC treated H_2_O. Next, 10 μg of total RNA was treated with RNase-free DNase (Qiagen) at 37 °C for 1 h and re-purified by a 3:1 phenol/chloroform extraction followed by an ethanol precipitation. From the purified RNA, 1 μg was used in the SYBR GreenER Two-Step qRT-PCR kit (Invitrogen) for first strand cDNA synthesis and the quantitative real-time PCR (qRT-PCR). The effects of perinatal Pb^2+^ exposure on genes related to early neuronal development and the GABA-shift were evaluated through the expression of: Ca-β3, GABA_AR_-β3, NKCC_1_, KCC_2_, and GAD 80, 86, 65, and 67 isoforms.

**Results:**

Perinatal Pb^2+^ exposure significantly altered the GABA-shift neurodevelopmental GOI expression as a function of Pb^2+^ exposure and age across postnatal development. Dramatic changes were observed with Ca-β3 expression consistent with a Pb^2+^ competition with L-type calcium channels. By PND 22, Ca-β3 mRNA was reduced by 1-fold and 1.5-fold in PFC and HP respectively, relative to controls. All HP GABA-β3 mRNA levels were particularly vulnerable to Pb^2+^ at PND 2 and 7, and both PFC and HP were negatively impacted by Pb^2+^ at PND 22. Additionally, Pb^2+^ altered both the PFC and HP immature GAD 80/86 mRNA expression particularly at PND 2, whereas mature GAD 65/67 were most significantly affected by Pb^2+^ at PND 22.

**Conclusions:**

Perinatal Pb^2+^ exposure disrupts the expression of mRNAs related to the GABA-shift, potentially altering the establishment, organization, and excitability of neural circuits across development. These findings offer new insights into the altered effects Pb^2+^ has on the GABAergic system preceding what is known regarding Pb^2+^ insults unto the glutamatergic system.

## Background

Lead (Pb^2+^) is a well-established environmental neurotoxicant, which at low levels of exposure causes deleterious effects to neurodevelopment. The immature brain is particularly vulnerable to Pb^2+^ given that young mammals have a higher absorption of minerals than adults [1], as well as the fact that the blood brain barrier is still developing [2]. Pb^2+^ exposure dose-dependently induces brain region specific effects on transcriptome gene expression [3, 4]. Moreover, behaviorally naive rats exposed developmentally to Pb^2+^ show sex-based altered gene expression changes in the HP of aged rats [5], suggesting that perinatal exposure can disrupt genetic programs in the absence of sensory and behavioral experience. These studies are consistent with neurodevelopmental Pb^2+^ exposure altering later life cognitive outcomes damaging the prefrontal cortex (PFC) and hippocampus (HP) as a function of gender and time-period of exposure.

Notably, most studies investigating neurodevelopmental Pb^2+^ exposure restricted their focus to adult outcomes [3–8] warranting earlier investigation of the underlying mechanisms producing this developmental neuropathology. Interestingly, even low blood lead levels (BLLs) can produce frontoexecutive dysfunctions and neuropathologies in children, which persist across the lifespan [2, 9, 10]. This highlights the need for early biomarkers of neuropathological disease that can predict developmental Pb^2+^ exposure problems early in postnatal life and that can be tracked across the lifespan. For example, Pb^2+^ exposure alters the levels of hippocampal NMDA receptor mRNA transcription and translation with associated learning and memory defects in adult rodents [3–8].

It is likely that perinatal Pb^2+^ exposure alters the expression of additional genes related to synaptic connectivity and function given the widespread effect of Pb^2+^ on the neurodevelopmental transcriptome [3, 4]. One possible mechanism by which Pb^2+^ might affect neurodevelopment is during the “GABA-shift” [11]. The GABA-shift is a crucial neurodevelopmental event in which GABA is converted from an excitatory to an inhibitory neurotransmitter. The initial depolarizing effect of GABA is due to the high intracellular concentration of chloride ions during the early postnatal period. Thus, the GABA-shift is an important neurodevelopmental event that plays a crucial role in activating and wiring the neural circuitry necessary for lifelong learning and memory [11]. Alterations in the levels of genes that regulate the GABA-shift could result in developmental neuropathological disorders [12]. In particular, the beta-3 subunit of the L-Type voltage sensitive calcium channel (Ca-β3), has been shown to autoregulate its own channel activity through excitation-transcription coupling as a function of neuronal excitability [13]. Since the Ca-β3 subunit has been shown to be responsible for driving gene expression in neurons, especially in early development when most other neurotransmitter systems are either less expressed of functionally inactive, the Ca-β3 expression levels naturally occurring in development serve a unique role in regulating the dynamic function of neuronal activity. This Ca-β3 functional regulation of gene expression different from the L-Type voltage sensitive calcium channel alpha-1 subunit (Ca-α1) pore forming subunits, that regulate neuronal activity and less of gene expression [13, 14]. Moreover, Ca-α1 have been shown to be disrupted by Pb^2+^, yet less is known regarding its impacts on Ca-β3 and how Pb^2+^ may affect gene expression related to the GABA-shift. We therefore investigated the effects of perinatal Pb^2+^ exposure on the neurodevelopmental patterns of the genes regulating the GABA-shift in rat prefrontal cortex (PFC) and hippocampus (HP) during the time-frame spanning the GABA-shift. The genes of interest (GOI) were as follows: the beta-3 subunit of the L-Type voltage sensitive calcium channel (Ca-β3), the *γ-amino butyric acid* receptor A-beta-3 receptor subunit (GABA-β3); the Na^+^-K^+^-Cl^−^Cl^−^cotransporter (NKCC_1_); the K^+^-Cl^−^Cl^−^cotransporter potassium/chloride co-transporter-1 (KCC_2_); and glutamic acid decarboxylase (GAD) early 80/86 and late 65/67 isoforms. We found that perinatal Pb^2+^ exposure alters the expression of these genes in a way that could have an impact on the timing and magnitude of the GABA-shift.

## Method

### Subjects

One month prior to pairing 10–14 week old behaviorally naïve Long Evans Hooded rats (*N* = 12) purchased from (Taconic, NJ) were randomly selected for breeding from to establish an in-house breeding colony to be designated as either receiving Control or Perinatal Pb^2+^ treatments, respectively. A total of eight females (*n* = 4 Control and *n* = 4 Perinatal) and four males (*n* = 2 Control and *n* = 2 Perinatal) rats were used as breeders to serve as Dams and Sires. The Dam’s litters each ranged from *N* = 6–10 pups per litter (*M* = 8, *SD* = 2), irrespective of Pb^2+^ treatment, with a majority of males over females. Since there were more males in each of the litters than females, to best assess and equally control for Pb^2+^ − and litter-effects across all developmental time-points under study, female pups were excluded due to an insufficient sample size availability from the litter’s obtained. From the included pup litters, 24 experimentally naïve male Long Evans hooded rat pups (Control *N* = 12; Pb^2+^ *N* = 12) were sacrificed under the College of Staten Island IACUC approval procedures. Rats were maintained under controlled temperature (24 ± 1 °C) and humidity (55 ± 5%), on a 12 h:12 h light: dark reversed cycle.

### Experimental design and procedures

The breeders were paired as two female Dams with one male Sire for 3 weeks as a timed pregnancy, which was assessed upon observation of the Dam’s copulatory plug. Following the 3 weeks of pregnancy, the Dams were then separated into individual cages from the Sires. Once the pups were born, which was defined as postnatal day (PND) 0, male pups were randomly sampled by selecting one male pup from each litter at each developmental time-point (i.e.*,* PND 2, 7, 14, and 22) to control for any individual litter effects as an extraneous variable. Thus, for each developmental time-point an (*N* = 6) pups were sacrificed, brain regions of interest removed, and subsequently used for mRNA analysis.

### Materials and Pb^2+^ administration

Control Dams were administered Purina RMH 1000 chow (Dyets, Inc.) absent of any lead source ad libitium for the duration of the experiment. In contrast, Lead treated Dams were administered the same food with lead acetate that was commercially engineered within the Purina RMH 1000 chow (Dyets, Inc.) containing 30 g/kg maltose dextrin, 1.5 g/kg Pb^2+^ (C_2_H_3_O_2_)_2_, and 0.1 g/kg yellow dye], which reflected a 996 ppm lead acetate exposure. Thus, Dams were administered lead acetate through their only food source *ad libitium* from 2 weeks prior to pairing and continued throughout gestation until the sacrifice of their pups at each developmental time-point (i.e.*,* PND 2, 7, 14, and 22) defining a perinatal period of exposure (i.e.*,* − PND 34 to PND 22). At PND 0 when the pups were born, they were continually administered lead acetate via the Dam’s lactation as their source of Pb^2+^ exposure from PND 0 to PND 13. When the rat pups were able to each from the food hopper independently at PND 14 to PND 22, they then obtained Pb^2+^ exposure from two sources, both the Dam’s lactation and the food *ad libitium*.

### Blood lead level analyses

At the indicated PND of development time-point sacrifice, blood samples were collected with a 2 mL anti-coagulant EDTA coated syringes (Sardstedt, Germany), mixed to prevent coagulation, and then frozen at ^−^ 80 °C. Blood samples were sent out for commercial analysis by Magellan Diagnostics (North Billerica, MA) to determine the amount of lead in the blood by electrochemical anodic stripping voltammetry (ASV) to eliminate any potential for experimenter bias. Briefly, the ASV procedure lyses red blood cells (RBCs) so that Pb^2+^ are liberated. Then a negative electrochemical potential that was pre-applied to the test sensor strip was used to attract and aggregate the Pb^2+^ ions as a reduction step. Sequentially, an oxidation reaction was used to strip the aggregated Pb^2+^ ions by reversing the sample to a positive electrochemical potential and the amount of Pb^2+^ was then determined from the sample volume and calculated as the area under the curve. Therefore, the blood lead levels (BLLs) were processed using the ASV method by taking 50 μL of whole blood mixed with 250 μL of hydrochloric acid solution (0.34 M) and then applying the final mixture to the lead sensor strip and inserted into an ESA LeadCare II Blood Lead Analyzer system (Magellan Diagnostics, North Billerica, MA). After 3 min, the BLLs were reported from the instrument in μg/dL with lower sensitivity cut off value of 3 μg/dL and a high sensitivity cut off value of 65 μg/dL, with a standard error sensitivity detection level of ±1.5 μg/dL. If a BLL value were to occur below the lower limit, it was reported as < 3 μg/dL and if a BLL value were to occur above the higher limit, it was reported as > 65 μg/dL. Once all samples were commercially processed, the BLL data reports were generated and sent back to the researchers.

### Tissue sample collections

At PND 2, 7, 14 and 22 rats were randomly sampled from three different litters for each treatment condition, sacrificed, and their frontal cortices and hippocampi extracted under two-minutes, frozen, and stored at ^−^ 80 °C.

### RNA preparation

The PFC and HP total RNA was prepared using TRIzol Reagent (Invitrogen) consistent with our prior work [15, 16]. Briefly, 50–60 mg of wet brain tissues were homogenized in 1 mL of TRIzol reagent per 100 mg of tissue using a glass-Teflon homogenizer. Post-centrifugation, RNA was extracted with chloroform and precipitated with isopropyl alcohol. RNA samples were then re-suspended in 100 μL of DEPC treated H_2_O.

### Preparation of cDNA and quantitative real-time PCR analysis

Next, 10 μg of total RNA was treated with RNase-free DNase (Qiagen) at 37 °C for 1 h and re-purified by a 3:1 phenol/chloroform extraction followed by an ethanol precipitation. From the purified RNA, 1 μg was used in the SYBR GreenER Two-Step qRT-PCR kit (Invitrogen) for first strand cDNA synthesis and the quantitative real-time PCR (qRT-PCR). The qRT-PCR primers are listed in Table 1. All experiments were performed in triplicates and repeated twice for each experiment. All qRT-PCR reactions were analyzed through an ABI 7500 sequence detection system (Applied Biosystems).

**Table 1:** Oligonucleotides used in the real-time qRT-PCR reactions

GAPDH *ORF*	
Forward primer	5’-ACAGGGTGGTGGACCTCATG-3′
Reverse primer	5’-GTTGGGATAGGGCCTCTCTTG-3’
GABA_A_ β3 *ORF*	
Forward primer	5’-CCACGGAGTGACAGTGAAAA-3’
Reverse primer	5’-CACGCTGCTGTCGTAGTGAT-3’
CACNB β3 *ORF*	
Forward primer	5’-TGGATCGGGAGGCTAGTGAA-3’
Reverse primer	5’-CACGCTGCTCGTAGTGAT-3’
NKCC_1_ *ORF*	
Forward primer	5’-ATGAGTCTTCCAGTTGCCCG-3’
Reverse primer	5’-GCAACGTGTCCATGTGCTTT-3’
KCC_2_ *ORF*	
Forward primer	5’-GGACCCCCGCATACAAAGAA-3’
Reverse primer	5’-CCTCCAGACCTTGTGGTGAC-3’
GAD 80 *ORF*	
Forward primer	5’-AGTGTGGCCTCCAGAGGTTC-3’
Reverse primer	5’-TGGATATGGCTCCCCCAGGAG-3’
GAD 86 *ORF*	
Forward primer	5’-TGGCCTCCAGAGGTGATGGT −3’
Reverse primer	5’-TGGATATGGCTCCCCCAGGAG −3’
GAD 65 *ORF*	
Forward primer	5’-GGCTCTGGCTTTTGGTCCTTC -3’
Reverse primer	5’-TGCCAATTCCCAATTATACTCTTGA −3’
GAD 67 *ORF*	
Forward primer	5’-GCTGGAAGGCATGGAAGGTTTTA-3’
Reverse primer	5’-AATATCCCATCACCATCTTTATTTGACC -3’

### Target DNA sequence estimations

Target DNA sequence quantities were estimated using Zhang et al. [15] and Shen et al. [16] procedures. Briefly, the target DNA sequence quantities were estimated from the threshold amplification cycle number (*C*_*T*_) using a 7500 Sequence Detection System Software. The Δ*C*_*T*_ values were obtained by subtracting the respective GOI primer *C*_*T*_ values from the corresponding housekeeping gene *glyceraldehyde 3-phosphate dehydrogenase* (GAPDH) *C*_*T*_ values to normalize the cDNA differences. Relative mRNA levels were expressed as 2^(−Δ *CT)*^ X 100% of GAPDH. Data were then transformed using a Log_10_ calculation to assess relative fold changes across all GOIs under investigation to characterize their neurodevelopmental expression patterns as a function of age, treatment, and brain region.

### Statistical analyses

Data were analyzed with IBM SPSS version 24. A multi-factorial *ANOVA* with a *Tukey’s HSD* post hoc comparisons test and a partial Eta squared (${\eta}_p^2$) were used to assess *Age, Treatment,* and *Age X Treatment* interaction effects for each GOI per brain region. Significance levels were set at α = 0.05 and a CI of 95%. Data are presented as the mean ± SEM for both BLLs and all mRNA comparisons.

## Results

### Pup and dam BLLs

BLLs were determined from the pups and dams simultaneously. The average pup BLL was 44.67 μg/dL (*SEM* = 0.48; *n* = 4), 36.00 μg/dL (*SEM* = 0.63; *n* = 4), 30.33 μg/dL (*SEM* = 0.67; *n* = 4), and 37.33 μg/dL (*SEM* = 0.58; *n* = 4) for PND 2, 7, 14 and 22 respectively. The average dam BLL was 37.00 μg/dL (*SEM* = 0.58; *n* = 4), 41.33 μg/dL (*SEM* = 0.72; *n* = 4), 39.33 μg/dL (*SEM* = 0.93; *n* = 4), and 43.67 μg/dL (*SEM* = 0.86; *n* = 4) for dams at PND 2, 7, 14 and 22, respectively. All control dams and pups were Pb^2+^ negative. Notably, the Dam’s nor the pup’s body weights were not significantly different from one another at each developmental time-point as a function of Pb^2+^ lead treatment (data not shown).

### Pb^2+^ effects on Caβ3 and GABA-β3 mRNA

Postnatal changes in the expression of the potassium/chloride co-transporter (KCC_2_) and the sodium/potassium chloride co-transporter (NKCC_1_) regulate the GABA-shift from excitatory-to-inhibitory neurotransmission. Perinatal Pb^2+^ exposure could disrupt this shift by its action on L-type calcium channels and downstream effects on KCC_2_ and NKCC_1_ expression levels. We therefore compared pup brain mRNA expression levels of Ca-β3, NKCC_1_, KCC_2_, GABA-β3, GAD 80/86, and 65/67 from control and maternally Pb^2+^ exposed rats at various time points after birth.

**Fig. 1:**
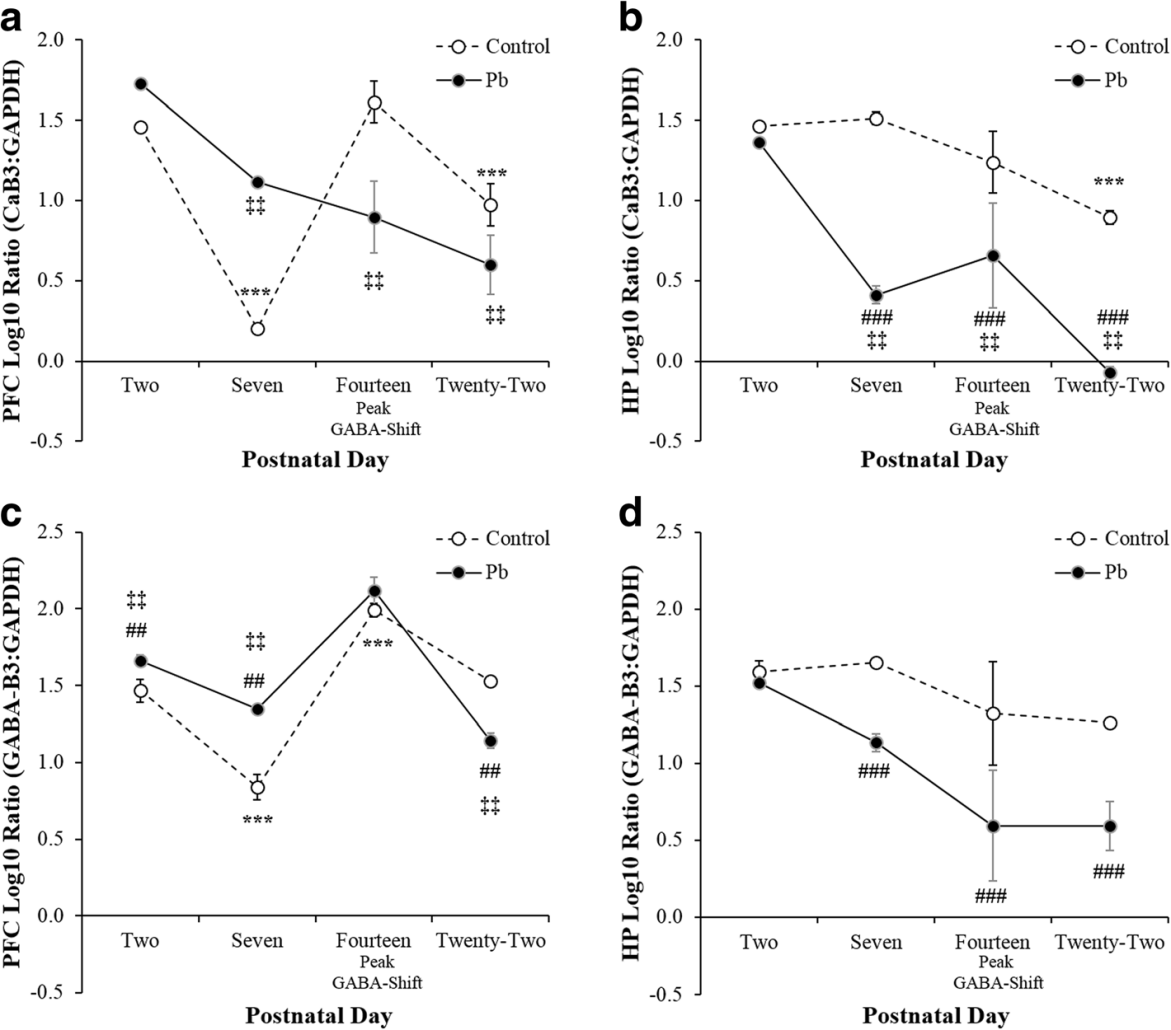
Illustrates the PFC (**a** & **c**) and HP (**b** & **d**) neurodevelopmental expression of Ca-β3 **(upper panel)** and GABA_AR_-β3 **(lower panel)** mRNA between Control and Pb^2+^ exposed rats. Perinatal Pb^2+^ exposure alters the expression of Ca-β3 mRNA with increased vulnerability in the PFC at PND 7 and 14, and in the HP at PND 7 and 22. Pb^2+^ exposure resulted in an altered regulation of GABA_AR_-β3 mRNA expression at PND 7 and 22 in the PFC and a down regulation from PND 7–22 in the HP. Data are presented as ± SEM and *Tukey’s* post hoc analyses are denoted as a significant difference in Control rats (*p* < 0.05*, *p* < 0.01**, *p* < 0.001***) as a function of *Age*, and denoted as a significant difference between Pb^2+^ vs. Control (*p* < 0.05^#^, *p* < 0.01^##^, *p* < 0.001^###^) as a function of *Age* and an *Age X Treatment interaction* for each developmental time-point (*p* < 0.05^‡^, *p* < 0.01^‡‡^, *p* < 0.001^‡‡‡^)

**Fig. 2:**
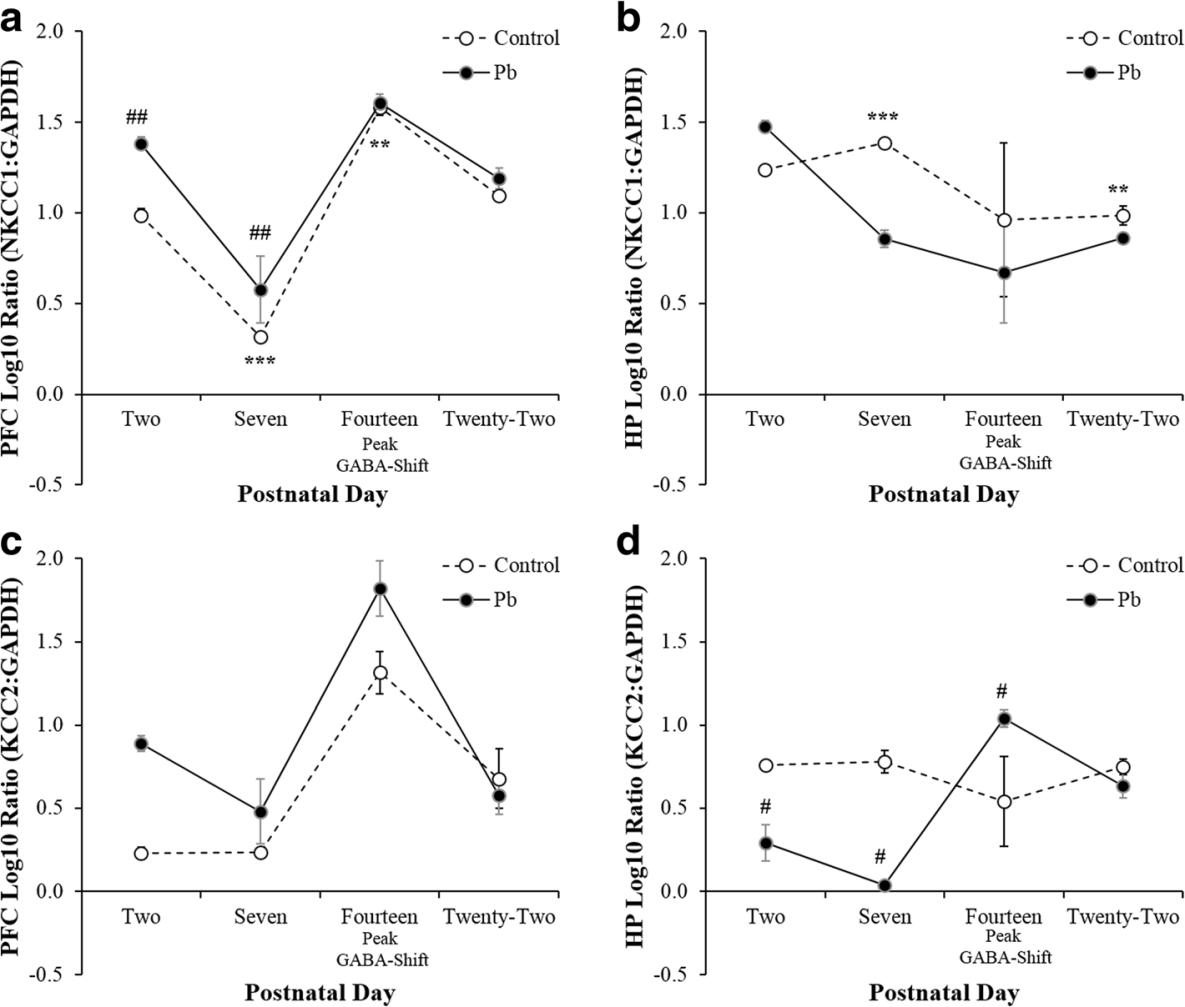
Illustrates the PFC (**a** & **c**) and HP (**b** & **d**) neurodevelopmental expression of NKCC_1_ **(upper panel)** and KCC_2_ **(lower panel)** mRNA between Control and Pb^2+^ exposed rats. Perinatal Pb^2+^ exposure resulted in an upregulation of NKCC_1_ in the PFC at PND 2, whereas in the HP NKCC1 was slightly upregulated at PND 2 and down regulated at PND 7. At PND 2 and 14 KCC_2_ was upregulated by Pb^2+^ exposure. However, Pb^2+^ exposure caused a down regulation of KCC_2_ mRNA expression at PND 2 and 7, followed by an upregulation at 14 in the HP. Data are presented as ± SEM and *Tukey’s* post hoc analyses are denoted as a significant difference in Control rats (*p* < 0.05*, *p* < 0.01**, *p* < 0.001***) as a function of *Age*, and denoted as a significant difference between Pb^2+^ vs. Control (*p* < 0.05#, *p* < 0.01##, *p* < 0.001###) as a function of *Age* and an *Age X Treatment interaction* for each developmental time-point (*p* < 0.05^‡^, *p* < 0.01^‡‡^, *p* < 0.001^‡‡‡^)

In PFC, Ca-β3 was dynamically regulated during postnatal development in control animals, decreasing in expression by nearly 1.5 fold as an *Age* effect between PND 2 and 7 *F*_(3,20)_ = 24.51, *p* < 0.001***, ${\eta}_p^2$ = 0.821 (Fig. 1a). Expression levels recovered between PND 7 and 14, and then decreased slightly at PND 22. Perinatal Pb^2+^ exposure completely blunted this regulation with Ca-β3 at similar levels initially at PND 2, with a gradual decrease in expression over the time course evidencing an *Age X Treatment* interaction *F*_(3,1,20)_ = 17.03, *p* < 0.001^‡‡‡^, ${\eta}_p^2$ = 0.762. Contrastingly, in HP, Ca-β3 mRNA exhibited an *Age* effect as a steady gradual decline in levels from PND 2 to 22 *F*_(3,20)_ = 17.46, *p* < 0.001***, ${\eta}_p^2$ = 0.766 (Fig. 2b). Pb^2+^ exposure decreased the expression levels of Ca-β3 at each time point with a significant *Treatment* effect *F*_(1,20)_ = 49.27, *p* = 0.001^###^, ${\eta}_p^2$ = 0.755 and an *Age X Treatment* interaction *F*_(3,1,20)_ = 5.28, *p* = 0.01^‡‡^, ${\eta}_p^2$ = 0.498. Thus, these results suggest that Pb^2+^ exposure causes the neurodevelopmental misregulation of Ca-β3 in both PFC and HP.

In PFC, GABA-β3 (Fig. 1c), similar to Caβ3, was dynamically regulated in control animals with increased expression at PND 2 and 14 and reduced expression at PND 7 and 22 as an *Age* effect *F*_(3,20)_ = 98.01, *p* < 0.001***, ${\eta}_p^2$ = 0.948. Treatment with Pb^2+^ *F*_(1,20)_ = 7.08, *p* < 0.02^#^, ${\eta}_p^2$ = 0.307 significantly altered this regulation in a manner distinct from that in the control pups and further evidenced an *Age X Treatment* interaction *F*_(3,1,20)_ = 19.89, *p* < 0.001^‡‡‡^, ${\eta}_p^2$ = 0.798. Although Pb^2+^ did exhibit a trend towards blunting the decrease in GABA-β3 expression from PND 2 to 7, this change was not as dramatic as seen with Caβ3 in PFC between these two time points. At PND 14, Pb^2+^ did not cause a further decrease in GABA-β3 as it did for Caβ3; indeed expression recovered in a manner similar to control animals. GABA-β3 mRNA levels showed a similar response to Pb^2+^ as Caβ3 in HP, levels were reduced at each time point examined (Fig. 1d) with a significant effect of *Age F*_(3,20)_ = 5.79, *p* < 0.01**, ${\eta}_p^2$ = 0.521 and a *Treatment* effect *F*_(1,20)_ = 14.52, *p* < 0.001^###^, ${\eta}_p^2$ = 0.476. Overall, the results show that Pb^2+^ treatment altered the expression levels of Caβ3 and GABA-β3 at many developmental time points in both PFC and HP.

### Pb^2+^ effects on NKCC_1_ and KCC_2_ mRNA

In control PFC, NKCC_1_ mRNA exhibited a biphasic regulation similar to that of Ca-β3 mRNA with downregulated expression as an *Age* effect *F*_(3,20)_ = 82.29, *p* < 0.001***, ${\eta}_p^2$ = 0.939, at PND 7 that recovered by PND 14 to approximately the same levels at PND 2 (Fig. 2a). In contrast, to Ca-β3 however, there was a significant *Treatment* effect *F*_(1,20)_ = 13.75, *p* < 0.01^##^, ${\eta}_p^2$ = 0.462 with a notable increase in NKCC_1_ mRNA at PND 2 in the Pb^2+^ treated animals. In HP, NKCC_1_ mRNA remained fairly constant, but evidenced a significant *Age* effect from PND 2 through 22 *F*_(3,20)_ = 3.40, *p* < 0.04*, ${\eta}_p^2$ = 0.389 and Pb^2+^ treatment did not alter this expression profile to a significant extent (Fig. 2b). KCC_2_ mRNA in PFC exhibited a pattern of regulation similar to that of NKCC_1_ in control animals and this was not significantly altered in Pb^2+^ treated animals (Fig. 2c). Contrastingly in HP, KCC_2_ mRNA was significantly downregulated in Pb^2+^ treated animals with an *Age* effect most notable at PND 7 *F*_(3,19)_ = 3.95, *p* < 0.03*, ${\eta}_p^2$ = 0.286, with a *Treatment* effect *F*_(1,19)_ = 6.00, *p* < 0.03^#^, ${\eta}_p^2$ = 0.441, and an *Age X Treatment* interaction *F*_(3,1,19)_ = 9.50, *p* < 0.001^‡‡‡^, ${\eta}_p^2$ = 0.655 (Fig. 2d). Overall, the results suggest a lesser, and perhaps indirect response, in mRNA regulation to Pb^2+^ for NKCC_1_ and KCC_2_ versus the significant direct changes in Caβ3 and GABA-β3 mRNA in Pb^2+^ treated animals.

### Pb^2+^ effects on GAD 80/86 and 65/67 mRNA

**Fig. 3:**
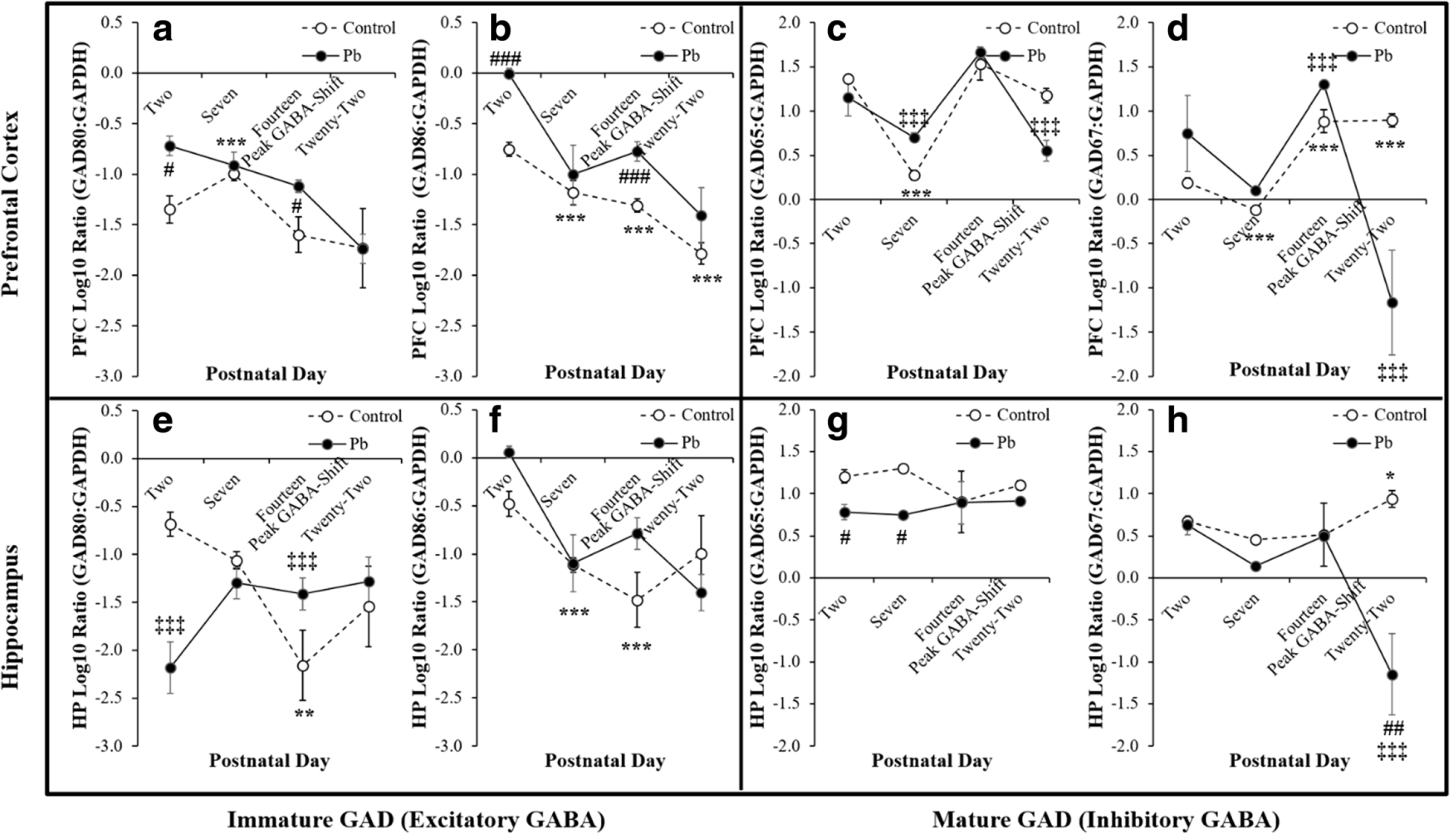
Illustrates the PFC (**a**-**d**) and HP (**e**-**h**) neurodevelopmental expression of GAD early 80 and 86 **(left panel)**, and late, 65 and 67 **(right panel)** mRNA between Control and Pb^2+^ exposed rats. Perinatal Pb^2+^ exposure disrupts early immature GABA by upregulating GAD 80 and 86 at PND 2 and 14 in the PFC, whereas GAD 80 was down regulated at PND 2 and upregulated at PND 14 in the HP. GAD 86 was upregulated at PND 2 and 14 in the HP. Pb^2+^ exposure resulted in altered mature GABA by upregulating GAD 65 at PND 7 and down regulating it at PND 22 in the PFC. Pb^2+^ exposure also caused a down regulation of GAD 67 in both the PFC and the HP at PND 22. GAD 65 was also down regulated at PND 2 and 7 in the HP. Data are presented as ± SEM and *Tukey’s* post hoc analyses are denoted as a significant difference in Control rats (*p* < 0.05*, *p* < 0.01**, *p* < 0.001***) as a function of *Age*, and denoted as a significant difference between Pb^2+^ vs. Control (*p* < 0.05#, *p* < 0.01##, *p* < 0.001###) as a function of *Age* and an *Age X Treatment* for each developmental time-point (*p* < 0.05^‡^, *p* < 0.01^‡‡^, *p* < 0.001^‡‡‡^)

In PFC GAD 80 mRNA expression revealed a significant effect of *Age F*_(3,20)_ = 7.78, *p* < 0.01**, ${\eta}_p^2$ = 0.593 and a *Treatment* effect *F*_(1,20)_ = 5.38, *p* < 0.05^#^, ${\eta}_p^2$ = 0.252 (Fig. 3a). We also observed similar outcomes in PFC GAD 86 mRNA with a significant effect of *Age F*_(3,20)_ = 19.09, *p* < 0.001***, ${\eta}_p^2$ = 0.782, and a *Treatment* effect *F*_(1,20)_ = 16.25, *p* < 0.001^###^, ${\eta}_p^2$ = 0.504 (Fig. 3b). In contrast, HP GAD 80 mRNA revealed only an *Age X Treatment* interaction *F*_(3,1,20)_ = 7.06, *p* < 0.01^‡^, ${\eta}_p^2$ = 0.570 (Fig. 3e). HP GAD 86 mRNA revealed only a significant effect of *Age F*_(3,20)_ = 8.41, *p* < 0.001***, ${\eta}_p^2$ = 0.612 (Fig. 3f).

In PFC GAD 65 mRNA revealed a significant effect of *Age F*_(3,20)_ = 34.02, *p* < 0.001***, ${\eta}_p^2$ = 0.864 and an *Age X Treatment* interaction *F*_(3,1,20)_ = 7.57, *p* < 0.01^‡‡^, ${\eta}_p^2$ = 0.587 (Fig. 3c). Also, PFC GAD 67 mRNA revealed a significant effect of *Age F*_(1,20)_ = 13.05, *p* < 0.001***, ${\eta}_p^2$ = 0.710 and an *Age X Treatment* interaction *F*_(3,1,20)_ = 16.21, *p* < 0.001^‡‡‡^, ${\eta}_p^2$ = 0.752 (Fig. 3d). In contrast, HP GAD 65 mRNA revealed only a significant effect of *Treatment F*_(1,19)_ = 5.90, *p* < 0.05^#^, ${\eta}_p^2$ = 0.282 (Fig. 3g). Also, HP GAD 67 mRNA revealed a significant effect of *Age F*_(3,19)_ = 3.95, *p* < 0.05*, ${\eta}_p^2$ = 0.442, *Treatment F*_(1,19)_ = 13.38, *p* < 0.001^###^, ${\eta}_p^2$ = 0.471, and an *Age X Treatment* interaction *F*_(3,1,19)_ = 8.95, *p* < 0.001^‡‡‡^, ${\eta}_p^2$ = 0.641 (Fig. 3h). The overall outcomes of Pb^2+^ effects on the mRNA of key genes responsible for the GABA-shift are summarized in Table 2.

**Table 2:** Summary of perinatal Pb^2+^ exposure results on developmental time-points altering the expression of genes related to the GABA-shift, when compared to the Control group

	*PFC Pb* ^*2+*^ *Effects*				*HP Pb* ^*2+*^ *Effects*			
*Genes*	*PND 2*	*PND 7*	*PND 14*	*PND 22*	*PND 2 PND 7*		*PND 14*	*PND22*
Caβ3	↑	↑	↓	↓	↔	↓	↓	↓
	n/s	‡‡	‡‡	‡‡	n/s	‡‡	‡‡	‡‡
						###	###	###
GABA-β3	↑	↑	↔	↓	↔	↓	↓	↓
	‡‡	‡‡	n/s	‡‡	n/s	###	###	###
	##	##		##				
NKCC1	↑	↑	↔	↔	↑	↓	↔	↓
	##	##	n/s	n/s	n/s	n/s	n/s	n/s
KCC2	↑	↔	↑	↔	↓	↓	↑	↔
	n/s	n/s	n/s	n/s	#	#	##	n/s
GAD-80	↑	↔	↑	↔	↓	↔	↑	↔
	#	n/s	#	n/s	‡‡‡	n/s	‡‡‡	n/s
GAD-86	↑	↔	↑	↔	↑	↔	↑	↔
	###	n/s	###	n/s	n/s	n/s	n/s	n/s
GAD-65	↔	↑	↔	↓	↓	↓	↔	↔
	n/s	‡‡‡	n/s	‡‡‡	#	#	n/s	n/s
GAD-67	↑	↑	↑	↓	↔	↓	↔	↓
	n/s	n/s	‡‡‡	‡‡‡	n/s	n/s	n/s	‡‡‡
								###

*Note:* Difference in expression of mRNAs are summarized as (↑) = an increase in mRNA, (↓) = a decrease in mRNA, and (↔) = no difference in relative mRNA expression. *Tukey’s* post hoc analyses are denoted as a significant difference between Pb^2+^ vs. Control as a function of *Treatment* (*p* < 0.05^#^, *p* < 0.01^##^, *p* < 0.001^###^) and *an Age X Treatment* interaction (*p* < 0.05^‡^, *p* < 0.01^‡‡^, *p* < 0.001^‡‡‡^) for each developmental time-point, whereas (n/s) = not significant

## Discussion

### The GABA-shift is a critical Ca^2+^-dependent neurodevelopmental process that is altered by perinatal Pb^2+^ exposure

There are two Ca^2+^-dependent genes critical for activating the neurodevelopmental GABA-shift: the *Slc12a2 and Slc12a5* genes, which encode NKCC_1_ and KCC_2,_ respectively. This Cl^−^-cotransporter gene family is responsible for maintaining cell volume regulation, epithelial transport, and GABAergic circuitry [17, 18]. The latter sets the neurodevelopmental sequences for precise Ca^2+^ wave oscillations driving GABAergic GDPs [11, 12], which regulate Ca^2+^-dependent gene signaling [19]. In the present study, it was hypothesized that perinatal Pb^2+^ treatment during perinatal development in the rat model would disrupt Ca^2+^-dependent gene signals, causing altered PFC and HP mRNA neurodevelopmental expression patterns. The data suggest that the coordination of this critical neurodevelopmental process is examinable through Ca-β3, GABA-β3, and NKCC_1_/KCC_2_ mRNA expression patterns as a function of postnatal age (Fig. 1). Pb^2+^ altered PFC Ca-β3 mRNA expression through an upregulation at PND 7 and a down regulation at PND 14, whereas GABA-β3 mRNA expression was significantly upregulated at PND 7 and down regulated at PND 22. Contrastingly, Pb^2+^ down regulated HP Ca-β3 mRNA expression at PND 7 and 22, whereas GABA-β3 mRNA expression was down regulated at PND 7 and 22. Thus, in both the PFC and HP, these genes were differentially altered by gestational Pb^2+^ exposure. Interestingly, HP Ca-β3 and GABA-β3 mRNA expression were more sensitive to Pb^2+^ than the PFC. It remains to be determined whether different neurodevelopmental GABA-shift trajectories exist for other brain areas.

### Pb^2+^ differentially alters the NCKK_1_/KCC_2_ GABA-shift in the PFC & HP

Perinatal Pb^2+^ exposure differentially altered the normal age-dependent NKCC_1_/KCC_2_ mRNA expression pattern in the PFC and HP (Fig. 2). In the PFC, at PND 2 NKCC_1_ mRNA expression was significantly upregulated (Fig. 2a) and at PND 2 and 14 the KCC_2_ mRNA expression was significantly upregulated (Fig. 2c). Interestingly, in the HP NKCC_1_ mRNA expression was upregulated at PND 2 and down regulated at PND 7 (Fig. 2b). However, the HP KCC_2_ mRNA expression was down regulated at PND 2 and 7 then upregulated at PND 14 (Fig. 2d). The pattern of NKCC_1_/KCC_2_ mRNA expression was different between the PFC (i.e.*,* NKCC_1_ vulnerability) and the HP (i.e.*,* NKCC_1_/KCC_2_ vulnerability). Notably, the PFC and HP NKCC_1_/KCC_2_ mRNA expression returned to control levels at PND 22 (Fig. 2). These neurodevelopmental NKCC_1/_KCC_2_ mRNA alterations underlie a molecular basis for increasing brain excitability in response to Pb^2+^ exposure, by two potential mechanisms: 1) prolonging early GABAergic excitation into adulthood or 2) delaying the onset of the mature inhibitory GABAergic system. Our data suggests that different brain regions may have unique neurodevelopmental time courses of NKCC_1_/KCC_2_ expression patterns [19–24] that may prove useful in early molecular diagnostic testing in clinical neurotoxicology.

### GAD isoforms provide unique insight into Pb^2+^ alterations of neurodevelopment

The early GAD isoforms 80/86 regulating the immature GABAergic excitatory system occur in embryonic development, whereas the late GAD isoforms 65/67 regulating the mature GABAergic inhibitory system occur in gestation and persist across the lifespan [25, 26]. The PFC GAD 80 mRNA and 86 mRNA expression were significantly affected by Pb^2+^ with upregulations at PND 2 and 14 (Fig. 3a-b), whereas the HP GAD 80/86 mRNA expression were not significantly affected by Pb^2+^ treatment, yet showed a down regulation for GAD 80 at PND 2 (Fig. 3e-f). The PFC GAD 65/67 mRNA expression were significantly down regulated at PND 22 and GAD 65 was down regulated at PND 7 in response to Perinatal Pb^2+^ treatment (Fig. 3c-d). The HP GAD 80 expression was significantly down regulated at PND 2 (Fig. 3b). However, the HP mRNA expression for GAD 65 was down regulated at PND 2 and 7, whereas the GAD 67 mRNA expression was significantly down regulated at PND 22 (Fig. 3g-h). The data suggest that Pb^2+^ exposure disrupted GAD 80/86 expression in the PFC and the HP during the gestational period with persisting impacts that were observed at PND 2 and its later life relationship with GAD 65/67 at PND 22 (Fig. 3). The findings from the present study, offer a novel mechanism for evaluating GAD isoforms in conjunction with the NKCC_1_/KCC_2_ GABA-shift transporters in assessing developmental Pb^2+^ neurotoxicology. This mechanism may prove informative for screening other developmental neurotoxicants other than Pb^2+^.

### GABA-shift disruption and developmental neuropathology

In the mature brain, the two major neurotransmitters *γ-amino butyric acid* (GABA) and *glutamic acid* (Glutamate), balance neural excitability. However, the immature GABAergic system is initially excitatory prior to the functional activation of the glutamatergic system. Whereby this switch is neurodevelopmentally regulated by NKCC_1_/KCC_2_ expression and functional activation [11, 12]. Notably, NMDA_R_ perturbations induced by perinatal Pb^2+^ exposure are known to contribute to lifelong intellectual disability [27, 28], but occur following the GABA-shift. However, the present study argued that given the functional silence of the NMDA_R_ system prior to GABAergic-dependent GDP activation [11, 12], that the GABAergic system may be more vulnerable to gestational and the Glutamatergic system to postnatal Pb^2+^ exposures. Alternatively, glutamatergic NMDA_R_ disruption may be a secondary consequence of Pb^2+^ exposure following early disruption of GABAergic excitation, NKCC_1_/KCC_2_ transporters, and GAD 80/86 and 65/67 interrelated events. Altogether, these findings implicate that clinical assessment of BLLs in children within the first year of life may be useful in determining gestational and postnatal neurodevelopmental risks associated with the maturation of the GABAergic system. Additionally, early neurodevelopmental Pb^2+^ poisoning can disrupt the predetermined pattern of genetic events that promote adequate myelination and synaptogenesis, which is most critical in the child’s early years [29]. Early disruption of these predetermined genetic events can result in a child deviating, disassociating, of disrupting the nature of typical human development and its accompanying milestones [30]. Essentially, it can be argued that consistent and appropriate early Pb^2+^ detection in children’s BLLs may be a valuable predictor of an altered inhibitory neurobehavioral profile in the child. Therefore, further study is warranted to elucidate GABAergic neurodevelopmental outcomes in response to Pb^2+^ insult producing developmental critical periods susceptible for acquiring neuropathological conditions prior to functional activation and involvement of the Glutamatergic system.

## Conclusion

In summary, this study shows that perinatal Pb^2+^ exposure through parturition can cause GABAergic neurodevelopmental alterations in the GOIs patterns of expression that regulate the GABA-shift through disruption of L-Type VSCCs signaling. Such aberrant neural excitability may cause either activity-dependent delays or premature switches of the NKCC_1_/KCC_2_ transporters dysregulating the GABA-shift in neurodevelopment, which are critical for establishing appropriate GABAergic networks within and across brain regions [17–25, 31–34]. The PFC and the HP were selected since the HP has been the brain region specifically studied in association with the GABA-shift in neurodevelopment [11, 12] and less is known regarding the PFC. Further, within the brain the PFC, HP and the cerebellum are most vulnerable for lead-induced brain damage as each region accumulates more lead deposition than other brain regions in clinical studies of children [36]. Thus, since less the PFC and its relationship with the HP are vulnerable to Pb^2+^ exposure during critical stages of neurodevelopment and they regulate higher order cognitive processes regarding frontoexecutive functions in contrast to the cerebellum, the study revealed that perinatal lead exposure could alter the expression of mRNA from genes involved in the GABA-shift. The clinical implications of these findings suggest that early developmental Pb^2+^ exposure may significantly alter the brains GABAergic networks, which may in turn, alter the developmental time-course of expression of the maturing inhibitory system. Thus, further work is required in describing the extent to which these observed mRNA altered expression patterns relate with physiological and behavioral changes in the effected individual. Results from perinatal Pb^2+^ exposure animal models have shown consistently deficits of inhibitory regulated behaviors across the lifespan, corroborating with the findings presented in this study. Further, such GABA-shift alterations can perhaps induce an array of brain excitability problems, increasing the susceptibility risks for incurring a spectrum of developmental neuropathologies that will persist across the lifespan. It is important to note that the GABA-Peak-Shift (as noted on each of the graphs at PND 14 on the data presented herein) are based solely on observations of the HP and one must be cautious in assuming that all brain regions follow the same time-periods of peak GABA-shifting. As such, the data presented in this study suggest that the PFC may have its own unique peak GABA-shift time-period from that of the HP. Moreover, perinatal Pb^2+^ exposure alters the normal age-dependent trajectory of the GABA-shift GOIs differentially dependent upon the brain region. This suggests that each brain region may “shift” at distinct time-periods of development and may equally present with neurotoxicant susceptibilities resulting in developmental neuropathologies during these precise time-periods. As such, Pb^2+^ exposure competes with critical Ca^2 + −^dependent gene activity dysregulating the GABA-shift as a model of neurological disease [34, 36] consistent with reports by Khale et al. [21], and Hyde et al. [35].,Moreover, neurodevelopmental Pb^2+^ exposure in children lacks an early developmental behavioral signature, yet interestingly neurocognitive patterns of impairments can be assessed later in life under behavioral learning and memory conditions [34, 36]. Further, it has been shown that NMDA_R_ blockade by Pb^2+^ and MK-801 can directly impair the acquisition learning [37, 38], but MK-801 antagonism has also been shown to impede the expression of inhibitory learning across the lifespan [39]. This suggests that neurodevelopmental Pb^2+^ exposure may cause similar dysfunctions in the expression of GABAergic-dependent learning. Thus, perinatal Pb^2+^ exposure can produce either GABAergic neurodevelopmental delays or suppression of neurotypical developmental gene expression patterns in the PFC and HP which can contribute and/or establish intellectual disabilities across the lifespan.

## Abbreviations

ASV: Anodic stripping voltammetry
BLL: Blood lead level
Ca-β3: L-Type voltage sensitive calcium channel-beta-3 subunit
GABA-β3: γ-amino butyric acid A-beta-3 receptor subunit
GAD: Glutamic acid decarboxylase
GAPDH: Glyceraldehyde-3-phosphate dehydrogenase
HP: Hippocampus
KCC_2_: Potassium/chloride co-transporter
NKCC_1_: Sodium/potassium chloride co-transporter
PFC: Prefrontal cortex
PND: Postnatal day
RBC: Red blood cell
